# Necrotizing Mediastinal Mass and Cavitary Pneumonia From Probable Co-infection With Epstein-Barr Virus and Mycoplasma pneumoniae

**DOI:** 10.7759/cureus.89642

**Published:** 2025-08-08

**Authors:** Drew A Wells, Jaime Baeza, StefaniRae Brewer, Chinelo Animalu

**Affiliations:** 1 Pharmacy, Methodist University Hospital, Memphis, USA; 2 Internal Medicine, Methodist University Hospital, Memphis, USA; 3 Infectious Diseases, Methodist University Hospital, Memphis, USA

**Keywords:** cavitary pneumonia, epstein barr virus (ebv), immunocompetent, immunocompetent adult, mycoplasma pneumonia, outcome of co-infection

## Abstract

Mycoplasma pneumoniae (MP) is a bacterium commonly known to cause mild respiratory infections, especially in young children. Epstein-Barr virus (EBV) is a herpesvirus that causes infectious mononucleosis, typically a mild illness in younger individuals. However, in its severe form, EBV can cause pneumonia. Severe pneumonia resulting from co-infection with MP and EBV is rare, with few cases reported in the literature, mostly in the pediatric population.

We report a case of a 20-year-old man who presented with a one-week history of progressively worsening right-sided pleuritic chest pain, subjective fevers and chills, night sweats, and a productive cough with purulent sputum. An initial extensive infectious work-up was unremarkable. Computed tomography (CT) imaging of the chest revealed a heterogeneous, possibly necrotic, posterior mediastinal mass, part of which encased the right inferior pulmonary vein and cavitary pneumonia. A previous CT scan of the abdomen, which captured the lower mediastinum three weeks prior to presentation, did not show the presence of this mass, suggesting a bacterial origin.

There was no response to initial empiric antimicrobials, necessitating an escalation of the antimicrobial regimen. After extensive work-up, the patient was found to have positive Mycoplasma immunoglobulin M (IgM) and positive EBV; however, Mycoplasma PCR testing could not be obtained. The antibiotic regimen was modified to include azithromycin, resulting in marked clinical improvement. Repeat CT scans at two weeks and three months showed a reduction in the size of the necrotic mass. The patient was subsequently discharged home in stable condition and has remained well.

## Introduction

Mycoplasma pneumoniae (MP) is an atypical bacterium that causes upper respiratory tract infections and community-acquired pneumonia [1,2]. With the recent increase in MP infections, more case reports state a rare and severe infection resulting in cavitary pneumonia [1,3,4]. Co-infection with another respiratory virus or bacteria is rare in immunocompetent adults [5]. It is more notably recognized in children with approximately 60% of children with MP pneumonia having a co-infection [6]. Epstein-Barr virus (EBV) is one virus that can cause co-infection with MP [5]. MP and EBV co-infection in children has been shown to increase the risk for prolonged symptoms or severe complications [6]. Very little is known about the clinical implications of MP and EBV co-infection in immunocompetent adults. Therefore, we present an interesting case of an immunocompetent adult male patient with a mediastinal necrotic mass and cavitary pneumonia caused by probable co-infection with MP and EBV.

## Case presentation

**Figure 1:**
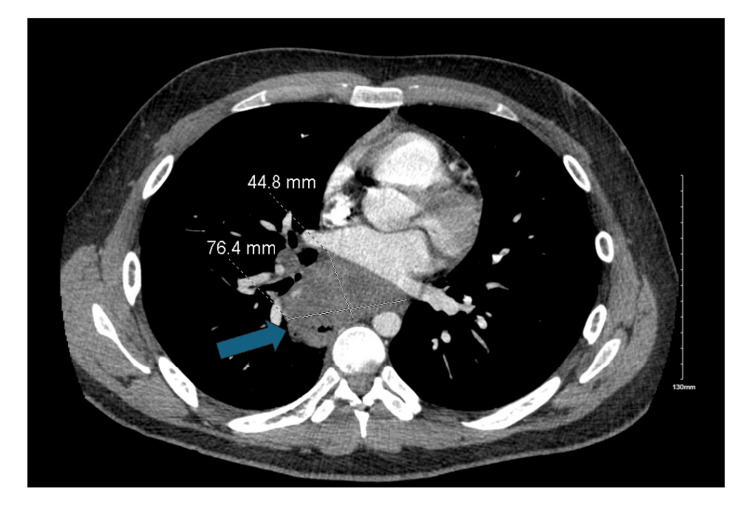
CT thorax on admission CT thorax with contrast on admission demonstrating a 7.6 cm necrotic posterior mediastinal mass partially encasing the right inferior pulmonary vein.

A 20-year-old man with a past medical history of gastroesophageal reflux and asthma presented to an outside hospital with a one-week history of progressively worsening right-sided pleuritic chest pain, subjective fevers, chills, night sweats, myalgias, and cough. Computed tomography (CT) imaging of the chest, abdomen, and pelvis demonstrated a heterogeneous, possibly necrotic, posterior mediastinal mass measuring up to 7.6 cm, part of which encased the right inferior pulmonary vein (Figure 1). 

**Figure 2:**
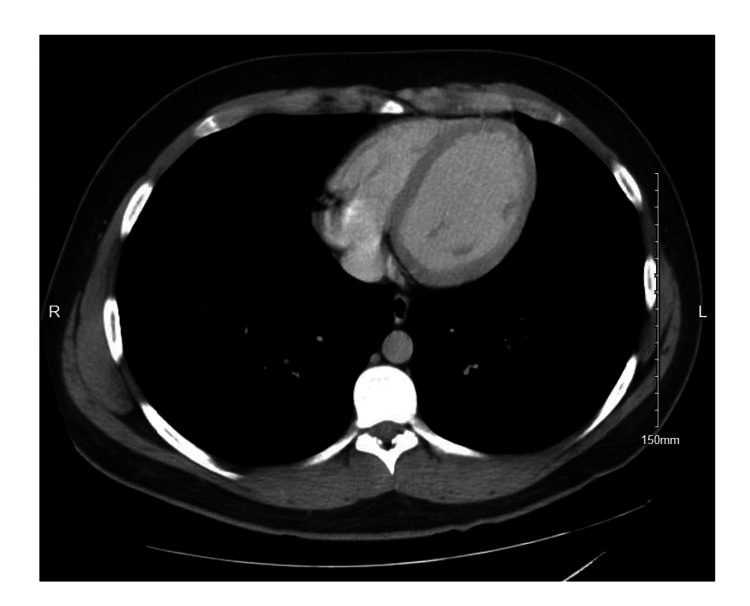
CT with contrast three weeks prior to admission CT abdomen/pelvis with contrast from three weeks prior to hospital admission demonstrating unremarkable exam.

Comparison with a prior CT abdomen three weeks prior to presentation did not show the mass (Figure 2). 

**Figure 3:**
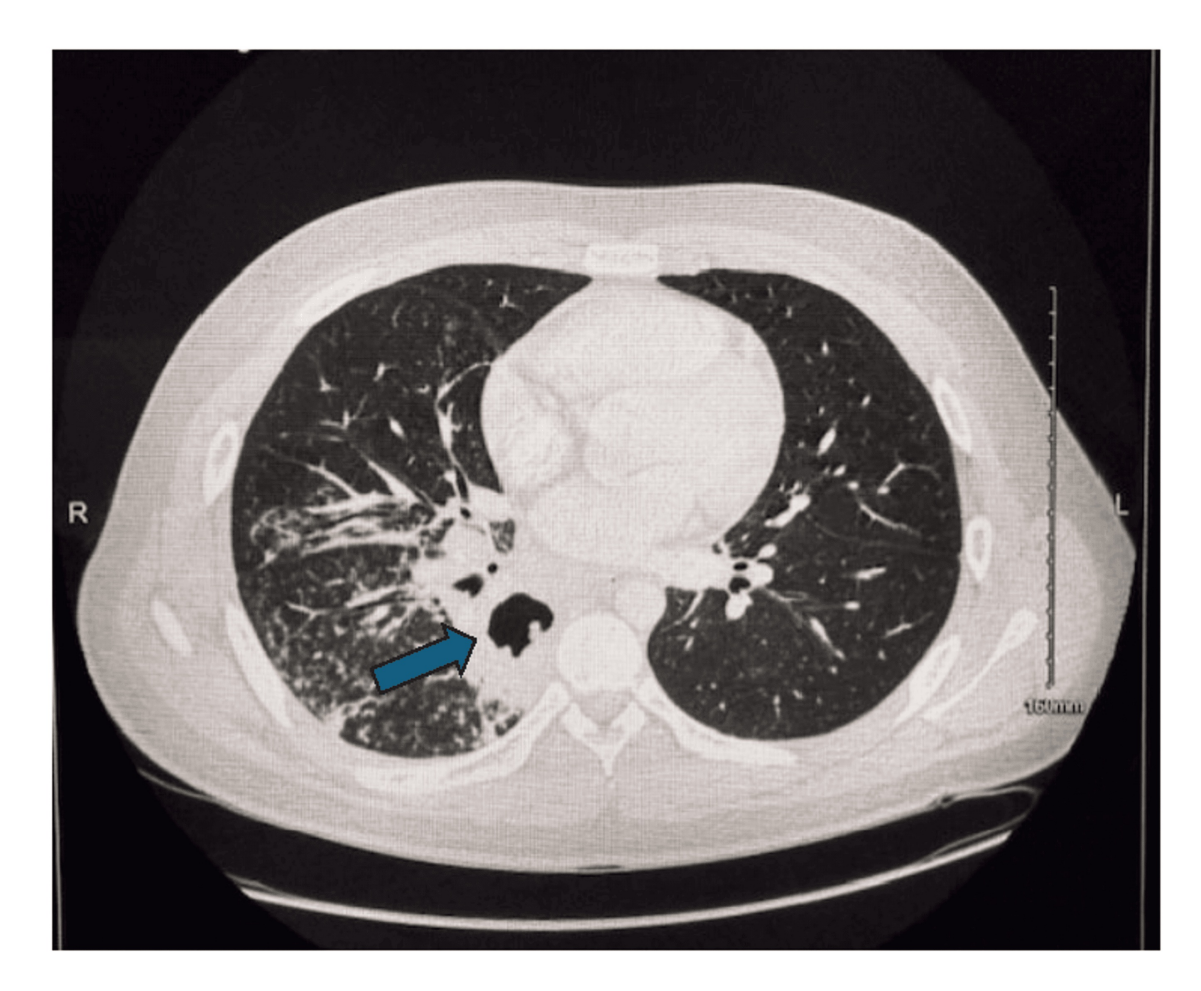
CT thorax two weeks later CT thorax with contrast two weeks after initial imaging demonstrating persistent but improved multifocal pneumonia with cavitation.

On admission, blood cultures were drawn and empiric antibiotic therapy with vancomycin and cefepime was initiated. Various infectious screenings were negative, including rapid respiratory viral panel, methicillin-resistant Staphylococcus aureus PCR nares, streptococcus NAAT, monospot test, HIV, hepatitis, and QuantiFERON gold. Sputum culture and blood cultures were also negative. Pertinent positive laboratory findings included an EBV PCR, KARIUS test (Prevotella and EBV), and MP IgM and IgG. On hospital day four, vancomycin and cefepime were discontinued and the patient was monitored. A bronchoscopy revealed a large subcarinal necrotic mass. Biopsies of the mass were negative for malignancy, granulomas, and fungus. Unfortunately, cultures of the mass were not obtained; thus, PCR testing from the sample could not be completed (i.e. MP PCR). On hospital day 6, he developed worsening leukocytosis (WBC 18.7 thou/mcL from 15.2 thou/mcL; reference range: 4.2 - 10.2 thou/mcL) and persistent high fever (oral temperature 39.2 ^o^C). Antibiotic therapy was changed to meropenem, linezolid, and azithromycin. The patient experienced significant improvement in symptoms with a notable decrease in WBC and resolution of fevers after a 10-day course. A repeat CT of the chest was obtained 14 days later which showed interval improvement in the multifocal pneumonia, with decreased volumes of infiltrates in the right and left lungs along with decreased density of consolidation and decreased size of cavitations (Figure 3). 

**Figure 4:**
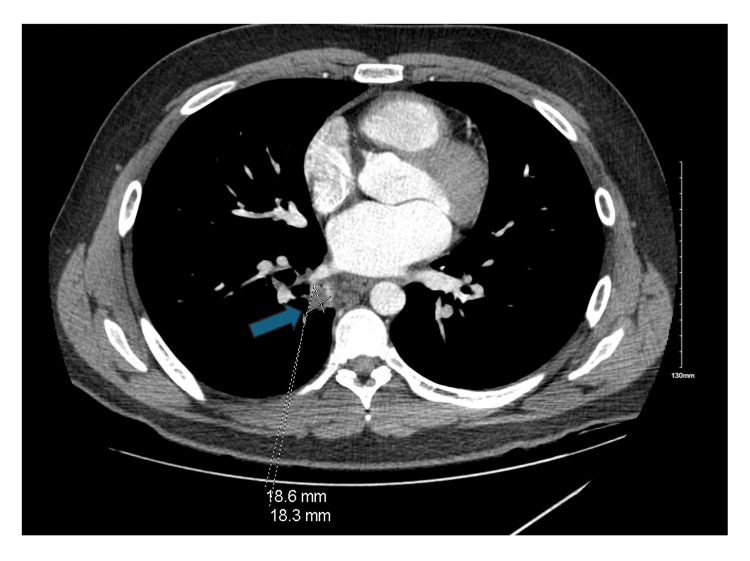
CT thorax three months later CT thorax with contrast three months after initial imaging demonstrating a significant decrease in the size of the cavitary lesion (1.8 cm) in the medial aspect of the right lower lobe.

Repeat CT imaging three months later revealed a significant decrease in the size of the cavitary lesion (1.8 cm) in the right lower lobe (Figure 4) and the patient was reported to be well. 

## Discussion

This case highlights a suspected case of MP and EBV co-infection in an immunocompetent adult male, leading to cavitary pneumonia and a necrotic mediastinal mass. Co-infection with EBV and MP is associated with longer duration of symptoms, elevated inflammatory markers, and increased risk of unresponsive/refractory pneumonia, which was seen in this case [2]. Cunha et al. reported a case in an immunocompetent 20-year-old female patient who presented with classic EBV infectious mononucleosis but was experiencing unexplained hypoxia and dyspnea [7]. Diagnostic testing revealed positive MP IgM and IgG titers. However, there was diagnostic uncertainty as to whether the degree of pulmonary involvement was due to EBV or MP. The patient was treated with a two-week course of levofloxacin and demonstrated improvement [7]. EBV pulmonary involvement has been reported but is rare [8]. Our patient did not display classic symptoms of EBV; rather, it was found by further diagnostic testing through Karius testing. EBV reactivation in this case could have occurred via several pathways, including enhanced immune modulation and cytokine dysregulation (i.e., interleukin-2), suppression of T-cell response leading to more pathological manifestations, and reactivation triggered by MP infection; however, there is limited evidence on the clinical impact the reactivation has in various clinical scenarios [2,9].

The initial imaging and diagnostic studies did not reveal the full extent of the pathology or provide diagnostic certainty. The presence of positive MP IgM in this patient provided serologic evidence of probable MP infection; however, certainty is limited by the lack of titers and PCR testing. Direct PCR testing, the gold standard, would have been highly sensitive and specific, but the lab was unable to perform the test due to a lack of respiratory sample during bronchoscopy [10,11]. Our case utilized Karius testing for diagnostic clarity. Karius testing in clinical practice is still novel and evolving. Karius testing, a commercially available microbial cell-free DNA assay, detects and quantifies microbial DNA directly from plasma, offering a non-invasive, broad-spectrum diagnostic tool that can identify over 700 pathogens, including bacteria, viruses, fungi, and parasites [12]. In the context of MP infection, Karius testing has demonstrated the ability to detect Mycoplasma species in plasma samples, even in cases where conventional diagnostics may be inconclusive [13,14]. While Karius testing offers rapid turnaround (median ~26 hours) and high analytical sensitivity, it may have reduced sensitivity for localized infections (i.e., confined to the respiratory tract) [12]. Thus, while the Karius test can be a powerful adjunct in the diagnostic workup of MP, especially in complex or refractory cases, it should not replace PCR-based respiratory testing, which remains the gold standard for direct detection of MP in respiratory specimens [11].

The primary treatment options for MP infection include macrolides, fluoroquinolones, and tetracyclines [15-17]. In this case, the patient did not receive any antibiotics with activity against MP until later in the hospital course. Given the patient’s deleterious condition and the initially unidentified source of infection, the escalation to linezolid, meropenem, and azithromycin was justified by the patient’s worsening clinical status, including persistent fever and leukocytosis, in the context of necrotizing pneumonia. Notably, the patient’s condition began to improve following escalation of therapy. While the clinical response is likely attributable, at least in part, to the macrolide therapy, it is important to acknowledge the confounding effect of concurrent linezolid and meropenem. It is possible given the lack of respiratory sample cultures that other bacterial pathogens could have been present that responded to treatment with linezolid and/or meropenem. However, the lack of improvement during the initial course of vancomycin and cefepime, both of which offer broad coverage but lack activity against atypical pathogens, further supports the role of azithromycin in the patient’s recovery.

Despite the uniqueness of our case, there are notable limitations. First, the lack of diagnostic certainty, including biopsy with gram stain, culture, PCR testing for MP specifically, and lack of MP titers, limited the diagnostic certainty of this case. Given the lack of PCR identification of MP, the diagnosis of probable co-infection was solidified based on clinical response to macrolide therapy in the setting of positive MP antibodies. While the authors acknowledge this is not confirmatory of co-infection, the limited reported information to date regarding co-infection, or the possibility, in the immunocompetent population makes this case insightful. Additionally, the use of broad-spectrum antibiotic therapy could suggest that another bacterial pathogen that was not isolated with microbiologic data could be causative of the pneumonia. Second, the presence of EBV on Karius testing is non-specific and hard to quantify the impact on the progression of this patient’s clinical course. While these pitfalls exist, the data presented with this unique case paints a picture consistent with a cavitary pneumonia and progressive mediastinal mass in a patient with diagnostic evidence of possible MP and EBV co-infection. 

## Conclusions

Co-infection with MP and EBV in an adult, immunocompetent patient is rare. Despite the limitations in diagnostic certainty, our case highlights a probable co-infection complicated by cavitary pneumonia and mediastinal mass that improved after initiating a macrolide among other antibiotic therapies.
